# Pharmacogenomics of NSAID-Induced Upper Gastrointestinal Toxicity

**DOI:** 10.3389/fphar.2021.684162

**Published:** 2021-06-21

**Authors:** L. McEvoy, D. F. Carr, M. Pirmohamed

**Affiliations:** Department of Pharmacology and Therapeutics, University of Liverpool, Liverpool, United Kingdom

**Keywords:** pharmacogenomic, NSAID (non-steroidal anti-inflammatory drug), gastrointestinal toxicities, aspirin, adverse drug reaction

## Abstract

Non-steroidal anti-inflammatory drugs (NSAIDs) are a group of drugs which are widely used globally for the treatment of pain and inflammation, and in the case of aspirin, for secondary prevention of cardiovascular disease. Chronic non-steroidal anti-inflammatory drug use is associated with potentially serious upper gastrointestinal adverse drug reactions (ADRs) including peptic ulcer disease and gastrointestinal bleeding. A few clinical and genetic predisposing factors have been identified; however, genetic data are contradictory. Further research is needed to identify clinically relevant genetic and non-genetic markers predisposing to NSAID-induced peptic ulceration.

## Introduction

NSAIDs comprise a heterogeneous group of non-opioid drugs with effective analgesic, anti-inflammatory and antipyretic properties (Calatayud and Esplugues, 2016). They are employed in the treatment of acute and chronic pain conditions characterized by inflammation. While aspirin was used as an analgesic in the past, its main use nowadays is as an antithrombotic particularly at low doses, and for cancer prevention (Shaheen et al., 2002; Sandler et al., 2003; Bashir et al., 2019). Overall, NSAIDs are well tolerated, especially when used short-term (Ong et al., 2007), but because of the enormous usage globally, they are often implicated in adverse drug reactions.

NSAIDs, including low dose aspirin (LDA), are one of the most commonly prescribed classes of medication, accounting for approximately 5–10% of prescriptions globally (Onder et al., 2004). Two decades ago, >30 million people were estimated to take NSAIDs daily (Singh and Triadafilopoulos, 1999). Pharmacoepidemiological studies indicate that NSAID use is increasing. A 2010 National Health Interview Survey (CDC, United States) reported increases of 57 and 41% in aspirin and NSAID use, respectively, over 5 years (Zhou et al., 2014), but this may still be an underestimate given the wide availability of aspirin and ibuprofen as over-the-counter (OTC) formulations. Telephone surveys of United States OTC NSAID users found that drugs were often used/taken inappropriately with 26% of respondents exceeding recommended doses (Goldstein and Lefkowith, 1998; Wilcox et al., 2005; Goldstein and Cryer, 2015). In Germany, analgesic use significantly increased from 19.2% in 1998 to 21.4% in 2008–2011. This rise was found to be attributed exclusively to the use of OTC formulations increasing from 10.0 to 12.2% (prescribed analgesic use remained constant at 7.9%). Ibuprofen was most commonly used, followed by aspirin (Sarganas and et al., 2015). Higher frequencies of ibuprofen use have also been documented in Denmark (Olsen and et al., 2011) and Spain (Gómez-Acebo and et al., 2018). In contrast, however, diclofenac was reported as the most frequently used NSAID (followed by ibuprofen) in a study across 15 countries (Australia, Bangladesh, Canada, China, China (Hong Kong), England, Indonesia, Malaysia, New Zealand, Pakistan, Philippines, Singapore, Taiwan, Thailand, and Vietnam) (McGettigan and Henry, 2013). The trend for increasing OTC analgesic use has also been echoed in Australia. Between 2001 and 2009, there was a 15% increase in the use of ibuprofen, naproxen and diclofenac (Stosic et al., 2011). In general practice, NSAID use by the elderly is prolific, reported at 96% (96% in males, 96.7% in females) in patients >65 years (Pilotto et al., 2003).

NSAIDs are responsible for ∼30% of ADR hospitalisations (Pirmohamed et al., 2004); cardiovascular, gastrointestinal (GI) and renal complications are associated with their use (Bhala et al., 2013; Szeto et al., 2020). Estimates suggest that 5,000–16,500 deaths in the United States and 400–1,000 deaths in the United Kingdom are a direct consequence of NSAID-induced upper GI ulceration and bleeding annually (Wolfe et al., 1999; Langman, 2001; Hawkey and Langman, 2003).

Genetic factors predisposing to NSAID-induced upper GI toxicity have been described, yet findings have been inconsistent and contradictory. This mini review discusses current literature and seeks to identify areas to focus collaborative efforts in the field.

## NSAID-Induced Upper Gastrointestinal Toxicity

The first serious NSAID-induced adverse event to be identified was upper GI injury (Walt et al., 1986; Langman, 1988). It is also recognized as one of the most predominant ADRs in the United States (Butt et al., 1988; Fries, 1996). NSAIDs and aspirin have now overtaken *Helicobacter pylori* as the principal cause of GI toxicity in western countries (Musumba et al., 2009; Bjarnason, 2013). “Gastrointestinal toxicity” refers to a collection of heterogeneous pathologies affecting various tissues and organs of the GI tract.

### Clinical Problem and Disease Burden

NSAID-related ADRs range from mild to severe and can result in death. Cryer (2004) conveniently stratified typical manifestations of NSAID-induced GI injury in to three tiers: dyspepsia; asymptomatic ulceration; and more serious complications (GI bleeding, perforation, obstruction, symptomatic ulceration) (Cryer, 2004). Although upper GI events are more common, the entirety of the GI tract may be affected (Maiden et al., 2005). Lower GI complications are not as well defined (Langman et al., 1985). In the large intestine, non-specific colitis, increased gut permeability, malabsorption and bleeding have been reported. Inflammatory bowel disease (IBD) (Bjorkman, 1998; Faucheron, 1999; Forrest et al., 1999) and diverticular disease (Campbell and Steele, 1991) may also be exacerbated by NSAIDs. In this article, we only focus on upper GI events.

Patient characteristics increasing the risk of NSAID-induced GI events have been identified (summarized in Table 1). These include: advanced age (Fries et al., 1991; Hernández-Díaz and Rodríguez, 2000; Russell, 2001; Sostres et al., 2013; Chi et al., 2018); *H. pylori* infection (Leontiadis and et al., 2007; Sostres et al., 2010); multimorbidity/comorbidity (Chi et al., 2018; Jankovic et al., 2009; Weil and et al., 2000; Kim, 2015); polypharmacy (Davies and Wallace, 1996) and concomitant medications (de Abajo et al., 1999; Silverstein et al., 2000; Sorensen et al., 2000; Garcia Rodriguez and Hernández-Díaz, 2001; Johnsen et al., 2001; Lazzaroni and Bianchi Porro, 2001; Laine et al., 2002; de Jong et al., 2003; Helin-Salmivaara et al., 2007; Lanas et al., 2007; Åhsberg et al., 2010; Masclee et al., 2013; Sostres et al., 2013; Anglin et al., 2014; Olsen et al., 2020). Studies have shown that the risk of NSAID-induced GI complications is dose-dependent (Silverstein and et al., 1995; Bombardier et al., 2000; Laporte et al., 2004; González-Pérez and et al., 2014; Figueiras and et al., 2016) and remains linear over time (Silverstein and et al., 1995; Bombardier et al., 2000; Rostom et al., 2007; Goldstein et al., 2011).

**TABLE 1:** Reported risk factors for developing NSAID-induced upper GI toxicity.

Patient characteristic	Description	References
**Patient factors**		
Advanced age	>60 years	Fries. (1996), Hernández-Díaz and Rodríguez, (2000), Chi et al. (2018)
	>70 years	Sostres et al. (2013), Russell. (2001)
Comorbidity/multimorbidity	Renal failure (receiving haemodialysis)	Jankovic et al. (2009)
	*Helicobacter pylori* infection	Leontiadis et al. (2007), Sostres et al. (2010)
	Diabetes mellitus	Weil and et al. (2000), Kim (2015)
	Cardiovascular disease	Chi et al. (2018)
Clinical history	Previous upper GI clinical event	Silverstein et al. (1995), Laine (2001), Laine (2006), Van Hecken et al. (2000), Sostres et al. (2013), Chi et al. (2018)
**Drug factors**		
Increased NSAID exposure	High dose/prolonged exposure NSAID therapy	Silverstein et al. (1995), Bombardier et al. (2000), Laporte et al. (2004)
		Garcia Rodriguez and Hernández-Díaz, (2001), Bhala et al. (2013), Lanas et al. (2015)
Polypharmacy	Polypharmacy	Davies and Wallace, (1996)
Concomitant drug use	Aspirin	Sorensen et al. (2000), Silverstein et al. (2000), Garcia Rodriguez and Hernández-Díaz, (2001), Åhsberg et al. (2010), Lazzaroni and Bianchi Porro, (2001)
	Non-aspirin antiplatelets	Sostres et al. (2013)
	Anticoagulants	Johnsen et al. (2001), Lanas et al. (2007), Olsen et al. (2020)
	Oral corticosteroids	Garcia Rodriguez and Hernández-Díaz, (2001), Laine et al. (2002), Masclee et al. (2013)
	Selective serotonin reuptake inhibitors	de Abajo et al. (1999), de Jong et al. (2003), Helin-Salmivaara et al. (2007), Anglin et al. (2014)

Abbreviations: GI, gastrointestinal; NSAID, non-steroidal anti-inflammatory drug.

Increased/prolonged exposure (habitual in chronic pain conditions through high-dose and multiple-NSAID use) elevates the risk and/or severity of toxicity (Garcia Rodriguez and Hernández-Díaz, 2001; Bhala et al., 2013; Lanas et al., 2015). Treatment guidelines recommend the lowest effective dose for the shortest period of time. Whilst it is recommended that long-term NSAID use should be avoided (Bhatt et al., 2008; Lanza et al., 2009), this is difficult in practice for many patients. Individuals with a past history of GI injury (including uncomplicated or complicated ulcers) are considered at the highest risk of complications (Sostres et al., 2013; Chi et al., 2018; Silverstein and et al., 1995; Van Hecken et al., 2000; Laine, 2001; Laine, 2006). COX-2 inhibitors (Coxibs) and gastroprotective therapies such as proton pump inhibitors (effective anti-secretory agents) help to mitigate the risk of GI injury (Gargallo et al., 2014; Melcarne et al., 2016).

Mild upper GI symptoms are reported by up to 40% of NSAID users (Larkai et al., 1989; Hirschowitz, 1994). The most clinically significant upper GI NSAID-induced ADRs are symptomatic and/or complicated peptic ulcer. Symptomatic peptic ulcer incidence rates in chronic NSAID users are between 2–4% annually (1–3% for serious ulcer/upper GI complications) (Cryer, 2004; Silverstein et al., 2000; Silverstein and et al., 1995; Bombardier et al., 2000; Blower and et al., 1997; Paulus, 1988), a threefold to fivefold increase compared to non-users (García Rodríguez and Jick, 1994; Gutthann et al., 1997; Hernández-Díaz and Rodríguez, 2000; Cryer, 2004; Gevers et al., 2014).

It is important to note that symptoms do not necessarily correlate with the severity of peptic ulcer disease and/or its complications (Sostres et al., 2013)—50–60% of patients can be asymptomatic (including having unremarkable endoscopies (McCarthy, 1989)) prior to developing potentially fatal NSAID-induced complicated ulcers (Armstrong and Blower, 1987). A prospective observational study found this figure to be as high as 80% (Singh and et al., 1996). In NSAID users who do develop lesions (endoscopically confirmed subepithelial hemorrhages, erosions, and ulcers), healing often occurs before symptoms manifest—“silent ulceration”—because of efficient gastroprotective mechanisms (Larkai et al., 1987; Lanas and Hunt, 2006; Sostres et al., 2013; Carr et al., 2017). Notwithstanding, peptic ulcer prevalence ranges from 15–40% in chronic NSAID users (McCarthy, 1989; Geis et al., 1991; Dajani and Agrawal, 1995; McCarthy, 1998). Gastric ulcer risk is slightly higher than duodenal ulcer risk (McCarthy, 1989; Larkai et al., 1987; McCarthy, 1998; Henry et al., 1993; Gabriel et al., 1991; Griffin and et al., 1991). Endoscopy remains the gold standard for PUD diagnosis (Dunlap and Patterson, 2019).

LDA is extensively used as prophylactic therapy against cardiovascular thrombotic events in high-risk individuals (Collaboration., 2002; Patrono et al., 2004). Throughout Europe and the United States, approximately 23% of the population use aspirin at least once per week (Larson et al., 2005). A prospective analysis of ADRs requiring hospitalization found that aspirin was the most commonly implicated, responsible for 18% of admissions; 74% of these patients were taking LDA. GI bleeding was the most common adverse event, observed in 72% of aspirin-related admissions (Pirmohamed et al., 2004). This is most likely to be due to aspirin-associated peptic ulceration (Niv et al., 2005). Generally, individuals on LDA therapy are older and multimorbid, increasing the likelihood of polypharmacy; some patients take both aspirin and NSAID(s) (Murray and et al., 1998) which is known to further raise the risk of GI toxicity and bleeding (Silverstein et al., 2000; Sorensen et al., 2000; Garcia Rodriguez and Hernández-Díaz, 2001). GI symptoms in chronic LDA therapy are challenging and are a reason for discontinuation of treatment and non/poor compliance (Cayla et al., 2012). Adverse events have been cited as responsible for LDA-discontinuation in almost 50% of patients (Herlitz et al., 2010). Discontinuation is associated with increased risk of new CV events (Biondi-Zoccai et al., 2006; Sostres and Lanas, 2011) and should only be advocated after sound clinical assessment when the risk of GI bleeding outweighs that of CV events.

NSAIDs differ in their propensity to cause upper GI injury based on their COX-1/COX-2 selectivity. Due to selective COX-2 inhibition, coxibs offer greater GI safety and are associated with a lower risk of upper GI injury and clinically significant ulcer complications compared to traditional NSAIDs (tNSAIDs) (Rostom et al., 2007; Chan et al., 2010). A meta-analysis by Castellsague et al. (2012) calculated the relative risk (RR) of upper GI complications as follows:

• RR <2 aceclofenac, celecoxib, ibuprofen.
• RR 2 to <4 diclofenac, ketoprofen, meloxicam, nimesulide, rofecoxib, sulindac.
• RR 4—5 diflunisal, indomethacin, naproxen, tenoxicam.
• RR >5 azapropazone, ketorolac, piroxicam (Castellsague et al., 2012).

Celecoxib has been shown to be associated with a lower risk of upper-GI events (Silverstein et al., 2000; García-Rayado et al., 2018) whilst celecoxib plus proton pump inhibitor is preferred over tNSAID (naproxen) plus proton pump inhibitor to reduce the risk of recurrent upper GI bleeding in individuals requiring concomitant aspirin and NSAID (Chan et al., 2017).

### Mechanisms of NSAID-Induced Gastrointestinal Toxicity

NSAID pharmacology, first described by Vane. (1971), is well documented (Vane, 1971). The cyclo-oxygenase (COX) pathway is responsible for the biosynthesis of prostanoids, including prostaglandins (PGs) and thromboxane (TX), from arachidonic acid (AA). Two clinically significant COX isoenzymes exist, COX-1 and COX-2, encoded by the genes *PTGS1* and *PTGS2,* respectively (Plaza-Serón et al., 2018). Prostanoids are pro-inflammatory mediators: PGs produced by COX-1 (and to a lesser degree, COX-2 (Wallace, 2008)) are essential in maintaining/regulating gastroprotection (Dickman and Ellershaw, 2004). Fundamentally, PG generation is forestalled by NSAIDs via reversible inhibition (except aspirin, an irreversible inhibitor (Funk et al., 1991; Catella-Lawson et al., 2001)) of COX isoenzymes, thereby decreasing inflammation.

NSAIDs are sub-divided based on COX-selectivity: tNSAIDs are non-selective, inhibiting both isoenzymes. COX-2 selective inhibitors (coxibs) are isoform-specific, designed to help mitigate the GI adverse events of tNSAIDs whilst retaining anti-inflammatory and analgesic activity. Inhibition of COX-2 is the desired therapeutic aim of NSAIDs. Upper GI toxicity is more common with tNSAIDs than with coxibs (Pilotto et al., 2005).

Under normal conditions, gut homeostasis is maintained. However, imbalance(s) in the gastroduodenal mucosal lining may result in GI injury. Protective factors comprise the gastric epithelial cells and hydrophobic mucus-bicarbonate bilayer, cellular repair, remodeling, restitution, and adequate blood supply–all of these are PG-regulated. Microvascular damage reduces gastric mucosal blood flow, an initial and crucial event in ulcer pathogenesis (Musumba et al., 2009; Bjarnason et al., 2018). The locale of greatest localized ischemia correlates with the site of most NSAID-induced ulceration, the gastric antrum (Musumba et al., 2009). Gastroprotective strategies can mitigate the risk of PUD, but will not be discussed further in this review: Wallace. (2008), Musumba et al. (2009), Bjarnason et al. (2018) provide excellent analyses (Wallace, 2008; Musumba et al., 2009; Bjarnason et al., 2018).

The pathogenesis of NSAID-induced GI injury is complex and multifactorial and can be divided into two broad areas: 1) Topical mechanisms (direct damage to GI mucosa). 2) Systemic mechanisms (COX-inhibition mediated).

1) *Topical effects—*NSAIDs can cause disturbances in the gastric mucosal epithelium initially through the development of erosions. NSAID toxicity is dependent on their physiochemical properties as lipid-soluble weak organic acids (pKa 3–5) (Brune et al., 1976; Brune and Graf, 1978; Rainsford and Whitehouse, 1980; Musumba et al., 2009; Bjarnason et al., 2018). Detergent properties allow interaction with surface membrane phospholipids, disrupting the mucosal barrier and provoking superficial injury (Lichtenberger, 2001). This permits NSAID to move from the lumen (low gastric pH measuring 1.5–3.5) into epithelial cells (pH neutral, 6.5–7.0), kickstarting disruption of the cellular metabolic pathways culminating in dysfunction, cytotoxic events and apoptotic pathway activation (Musumba et al., 2009; Handa et al., 2014).
2) *Systemic effects—*Robust gastric mucosal defence/repair is heavily dependent upon perpetual synthesis of COX-derived prostanoids. COX-1 is expressed in most tissues performing “housekeeping duties,” maintaining and regulating various physiological functions. In the GI tract, COX-1 is abundant, producing PGs and TXs involved in cytoprotection. COX-2 is expressed at low levels in the intact stomach, but rapidly induced by COX-1 inhibition or inflammatory stimuli/injury, and produced in vast quantities by cytokines and hormones (Rainsford, 2007; Ricciotti and FitzGerald, 2011; Wongrakpanich et al., 2018).

Classically, GI complications were primarily attributed to COX inhibition: decreased PG levels resulting in increased gastric acid secretion, suppressed mucus and bicarbonate secretion, decreased mucosal blood flow and decreased cell proliferation (Cohen, 1987; Wallace, 1992; Wallace, 2008) resulting in compromised mucosal defense and delayed healing. Selective COX-1 inhibition leads to suppression of mucosal PG production by 95–98% without observable inflammation or ulceration (Ligumsky et al., 1983; Ligumsky et al., 1990; Langenbach et al., 1995; Sigthorsson et al., 2002). Inhibition of COX-2 has yielded similar results (Morham et al., 1995; Sigthorsson et al., 2002). However dual inhibition of both isoenzymes by NSAIDs induces severe gastric lesions (Wallace et al., 2000; Gretzer et al., 2001; Tanaka and et al., 2001). Both COX isoenzymes are also important in ulcer healing (Tanaka et al., 2002; Schmassmann et al., 2006; To et al., 2001; Bhandari et al., 2005; Starodub et al., 2008; Chan and et al., 2005).

In isolation, topical mechanisms are unlikely to induce notable GI toxicity. In combination with systemic factors, however, NSAIDs provoke an imbalance that incites mucosal injury. Cellular damage translates into tissue damage. COX-1 inhibition dramatically suppresses gastroprotection leading to microvascular damage, ischemia, and decreased mucus production. A complex interplay between factors ignites a cascade, involving many mechanisms that disrupt gastroprotection, engendering ulcerogenic conditions.

## Genetic Risk Factors

It is well acknowledged that interindividual variability exists in response to drugs, including NSAIDs, which may at least be genetic in origin. Using conventional dosing regimens in analgesia, for example, some individuals will have inadequate pain control while others will encounter toxicity from the same dose (Kapur et al., 2014). Genetic variants affecting treatment outcomes can be categorized into two broad types: 1) genes affecting drug pharmacokinetics (PK), and 2) genes affecting drug pharmacodynamics (PD) (Stamer et al., 2010; Kapur et al., 2014). It should however be noted that given the different mechanisms of action and metabolic pathways, the PK and PD genetic variability cannot be generalized across all NSAID classes. Table 2 summarizes notable genetic risk factors for NSAID-induced upper GI toxicity.

**TABLE 2:** Notable genetic associations in NSAID-induced upper GI toxicity.

Genetic loci	Therapy	Phenotype	Association	*p*-value	Or (95%CI)	References
*CYP2C9*	NSAIDs—various	Endoscopically confirmed UGIB	CYP2C9*2 genotype (in heterozygosity or	*p* = 0.009	Crude OR = 1.92 (95% CI = 1.14–3.25)	Martínez et al. (2004)
			homozygosity) increases risk of GI bleeding			
*CYP2C9*	CYP2C9-metabolised	Endoscopically confirmed UGIB	Using CYP2C9*1/*1 wild type as control, significantly			Pilotto et al. (2007)
	NSAIDs		higher frequencies of bleeding observed in			
			CYP2C9*1/*3	*p* = < 0.001	OR = 12.9 (95% CI = 2.917–57.922)	
			CYP2C9*1/*2	*p* = 0.036	OR = 3.8 (95% CI = 1.090–13.190)	
			CYP2C9*3 allele carriers have significant risk of bleeding		Adjusted OR = 7.3 (95% CI = 2.058–26.004)	
*CYP2C9*	Non-aspirin NSAIDs	Endoscopically confirmed	CYP2C9*3 loss-of-function allele associated with acute	*p* = 0.0002	OR = 7.2 (95% CI = 2.6–20.3)	Carbonell et al. (2010)
		ulcers/bleeding erosions	UGIB in NSAIDs other than aspirin			
*CYP2C9*	CYP2C9-metabolised	Endoscopically confirmed	CYP2C9*3 variant increases risk of UGIB for defined daily doses >0.5		CYP2C9*3 OR = 18.07 (95% CI = 6.34–51.53)	Figueiras et al. (2016)
	NSAIDs	erosions/lesions/UGIB			Adjusted OR	
*CYP2C8 and CYP2C9*	CYP2C8/2C9-metabolised	Endoscopically confirmed UGIB	CYP2C8*3 and CYP2C9*2 exist in LD			Blanco et al. (2008)
	NSAIDs		Combined CYP2C8*3 + CYP2C9*2 genotype associated with increased risk of bleeding	*p* = 0.003	OR = 3.73 (95% CI = 1.57–8.88)	
*CYP2C19*	NSAIDs—various	Endoscopically confirmed UGIB	CYP2C19*17 associated with PUD, but not UGIB	*p* = 0.024	OR (additive model) = 1.47 (95% CI = 1.12–1.92)	Musumba et al. (2013)
			PUD distribution varied according to CYP2C19*17 genotype			
			*1/*1, 64.3%; *1/*17, 71.7%; *17/*17, 73.8%			
*COX-1*	Aspirin and ethanol		SNPs *A-842G* and *C50T* in complete LD			Halushka et al. (2003)
	pre-treatment		Heterozygous *A-842G*/*C50T* haplotype shows significantly	*p* = 0.01		
			greater PG H (2) inhibition			
*COX-1*	Not stated	Endoscopically confirmed	*A-842*/*C50T* polymorphism has lower (yet non-significant)		OR = 0.5 (95% CI = 0.18–1.34)	van Oijen et al. (2006)
		bleeding GU or DU	Risk of PU bleeding compared to wild type		Adjusted* OR = 0.75 (95% CI = 0.19–3.01)	
					Adjusted for: sex, age, smoking, NSAID/aspirin use, *H. pylori* infection	
*COX-2*	Aspirin (ASA)	Surgical or endoscopic UGIB	rs689466 T > C increases risk magnitude of UGIB in ASA users			Mallah et al. (2020)
		diagnosis	Variant carriers taking ASA v wild-type carriers NOT taking ASA	*p* = 0.0022	OR = 8.22 (95% CI = 2.14–31.59)	
			Variant carriers taking ASA v wild-type carriers taking ASA	*p* = 0.3036	OR = 2.98 (95% CI = 0.37–23.96)	
					OR adjusted as per Mallah et al. (2020)	

Abbreviations**:** ASA, (acetylsalicylic acid) aspirin; CI, confidence interval; DU, duodenal ulcer; GI, gastrointestinal; GU, gastric ulcer; LD, linkage disequilibrium; NSAID, non-steroidal anti-inflammatory drug; OR, odds ratio; PG H(2), prostaglandin H(2); PU, peptic ulcer; PUD, peptic ulcer disease; UGIB, upper gastrointestinal bleeding; SNP, single nucleotide polymorphism.

### Pharmacokinetic-Related Associations

Cytochrome P450 (CYP) gene polymorphisms are strongly associated with adverse drug reactions in general (Johansson and Ingelman-Sundberg, 2011; Sim et al., 2013). Collectively, CYP isoenzymes mediate ∼80% of phase I metabolism of clinically relevant drugs (Evans and Relling, 1999; Eichelbaum et al., 2006; Petrović et al., 2020), including NSAIDs (Blanco et al., 2005). Phase I oxidative metabolism precedes phase II conjugative metabolism (glucuronidation) driven by uridine 5′-diphosphate glucuronosyltransferases (UDP-glucuronosyltransferase, UGTs) (Kuehl et al., 2005; Monrad et al., 2014; Sandson, 2015), supplemented by sulfate conjugation (sulfotransferases) (Ulrich et al., 2006; Loetsch and Oertel, 2013). NSAID metabolism varies and this can be due to CYP polymorphisms, which have been implicated in NSAID-induced ADRs including PUD and UGIB. Studies have also demonstrated that GI damage is dose-dependent (Laporte et al., 2004; Figueiras and et al., 2016).

NSAID metabolism is specifically associated with the CYP2C subfamily (Ingelman-Sundberg et al., 2007; Musumba et al., 2013) (including the highly polymorphic CYP2C8, CYP2C9, and CYP2C19 isoforms), which make up 20% of hepatic CYP450 content and metabolize 25–30% of clinically used drugs. Polymorphisms in these genes may result in modified expression or functionality, correlating with altered metabolism and clearance which may affect bioavailability (Dai et al., 2001; Kirchheiner et al., 2002; Musumba et al., 2013; Krasniqi et al., 2016; Guengerich, 2020). Celecoxib, ibuprofen, lornoxicam and piroxicam are extensively (>90%) metabolized by CYP2C enzymes (Agúndez et al., 2009).

Estimates suggest that CYP2C9 is responsible for biotransformation and metabolic clearance in 15–20% of all phase I metabolized drugs (Goldstein and de Morais, 1994; Schwarz, 2003; Van Booven et al., 2010; Niinuma et al., 2014). CYP2C9 is involved as the main or secondary enzyme in the metabolism of most NSAIDs, including aceclofenac, aspirin, celecoxib, diclofenac, flurbiprofen, indomethacin, lornoxicam, meloxicam, naproxen, piroxicam, and tenoxicam (Davies et al., 2000; Rodrigues, 2005; Agúndez et al., 2009; Samer et al., 2013; Theken et al., 2020). With celecoxib, lornoxicam and piroxicam, CYP2C9 is the predominant enzyme, responsible for 90% of drug metabolism (Agúndez et al., 2009).

At least 62 variant alleles and multiple sub-alleles have been reported for *CYP2C9* (PharmVar (https://www.pharmvar.org/gene/CYP2C9) (PharmVar, 2017; Gaedigk et al., 2018; Theken et al., 2020) which vary in frequency ethnically, geographically and racially (Theken et al., 2020; Theken et al., 2020; Gene‐specific Information Tables for CYP2C9, 2020). The allelic variants CYP2C9*2 (rs1799853) and CYP2C9*3 (rs1057910) show high allele frequencies in human populations (García-Martín et al., 2006); the estimated prevalence in European populations is 14% for CYP2C9*2 and 8% for CYP2C9*3 (Lee et al., 2002; Xie et al., 2002; Sánchez-Diz et al., 2009). These variants, most notably CYP2C9*3, have been extensively studied and have been associated with decreased NSAID metabolism (Visser et al., 2005; García-Martín et al., 2006; Agúndez et al., 2009; Wadelius et al., 2009; Wang et al., 2011).

Carriers of low activity *CYP2C9* alleles could be at greater risk of GI toxicity due to increased NSAID exposure. Frustratingly, case-control studies investigating associations between CYP2C9*2 and *3 variants and NSAID-induced ADRs have often been contradictory. Several studies have reported no associations between *CYP2C9* variants and NSAID-induced PUD and/or UGIB (Martin et al., 2001; Van Oijen et al., 2005; Vonkeman et al., 2006; Lopezrodriguez et al., 2008; Ma et al., 2008; Musumba et al., 2013; Ishihara et al., 2014), while others have reported that *CYP2C9* variants predispose to PUD (Martínez et al., 2004; Pilotto et al., 2007), including the presence of a gene-dose effect (Figueiras and et al., 2016), and that there may be a combined effect of *CYP2C8*/*CYP2C9* variation (Blanco et al., 2005; Blanco et al., 2008).

Efforts to provide PGx-based guidance on NSAIDs recently culminated in the Clinical Pharmacogenetics Implementation Consortium (CPIC) guideline for *CYP2C9* and NSAIDs (Theken et al., 2020). The guideline categorizes *CYP2C9* alleles as follows: Normal function (wild type) CYP2C9*1, decreased function CYP2C9*2, *5, *8, *11, and no function CYP2C9*3, *6, *13 (Theken et al., 2020; Theken et al., 2020; Gene‐specific Information Tables for CYP2C9, 2020). *In vitro* and clinical studies have suggested that the “decreased-function” and “no-function” *CYP2C9* alleles are substrate-dependent. A meta-analysis of *CYPC9* variant alleles on NSAID exposure, based on predicted metaboliser phenotypes (poor metabolisers, intermediate metabolisers and normal metabolisers), showed that CYP2C9 poor metabolisers had decreased metabolic clearance, a prolonged plasma elimination half-life and increased plasma concentrations. Since NSAID-induced GI toxicity is known to be dose-dependent, the risk and severity of toxicity is likely to be increased (Bhala et al., 2013; Laporte et al., 2004; Figueiras and et al., 2016; Lanas et al., 2015). Hence, to mitigate the risk of PUD, dose-reduction, careful monitoring for toxicity/ADRs and alternative therapies, (e.g. non-CYP2C9-metabolized NSAIDs (such as naproxen)) should be used (Theken et al., 2020).

A subsequent systematic review and meta-analysis by Macías et al. (2020) also addressed discrepancies in previous studies. In an exhaustive study, they concluded: “There is a clear and consistent association of the development of GI adverse events with the *CYP2C9* genotype, and the association is slightly stronger in patients with GI bleeding.” Consistent with this, Theken et al. (2020), found that the associations were stronger in poor metabolisers (again based on metaboliser phenotypes) with a significant gene-dose effect. Furthermore, allele-specific analyses showed that CYP2C9*2 was a poor risk predictor (marginal effects) in contrast to CYP2C9*3, which clearly showed a highly significant association with increased risk of upper GI adverse events and GI bleeding (Macías et al., 2020). This supports the findings of Figueiras and et al. (2016) who demonstrated dose-dependency in NSAID-induced GI damage and the indication that the CYP2C9*3 allele can be used as a predictive UGIB risk marker for CYP2C9-metabolised NSAIDS (Figueiras and et al., 2016).

CYP2C8 plays an accessory metabolic role for certain NSAIDs including ibuprofen and diclofenac (Agúndez et al., 2009; Garciamartin and et al., 2004). CYP2C9*2 is in strong linkage disequilibrium (LD) with CYP2C8*3 (Speed et al., 2009). Findings however have again been conflicting: no association between CYP2C8*3 and UGIB was reported by Musumba *et al.* (Musumba et al., 2013) while others have reported a positive association with GI bleeding (Agúndez et al., 2009), including when combined with CYP2C9*2 (Blanco et al., 2005; Blanco et al., 2008).

CYP2C19 plays a minor role in NSAID metabolism. Interestingly, we showed that the CYP2C19*17 “gain of function” polymorphism was associated with PUD (Musumba et al., 2013), irrespective of the etiology of PUD. This may be related to the fact that CYP2C19 is involved in the metabolism of AA, and more extensive metabolism of AA may impair gastric mucosal defences. This is consistent with the fact that disrupted AA metabolism is involved in PUD pathogenesis (Musumba et al., 2009).

Thus, the role of the of the *CYP2C* gene locus in predisposing to PUD is complex and likely to vary with the NSAID given, its dose and the complement of SNPs the patient has at the *CYP2C* gene locus, including at *CYP2C9* (where low activity variants will increase exposure to NSAIDs) and the gain of function polymorphism at *CYP2C19* which increases the metabolism of gastro-protective AA.

### Pharmacodynamic-Related Associations

COX enzymes produce PGs from AA, playing vital roles in gastric defence. COX enzymes are the primary target of NSAIDs. There is potential for peptic ulcer pathogenesis to be influenced by functional polymorphisms in the COX-encoding genes (Agúndez et al., 2015). Individuals carrying reduced function COX-enzymes may be potentially susceptible to NSAID-induced peptic ulceration (Musumba et al., 2009).

*COX-2* single nucleotide polymorphism (SNPs) have been observed to affect responsiveness to celecoxib (Lee et al., 2017), but no association with NSAID-induced GI injury was reported. Studies of *COX-1* genetic polymorphisms and GI injury have also yielded contradictory findings (van Oijen et al., 2006; Arisawa et al., 2007). As such, there is insufficient evidence currently to determine whether the *COX* gene polymorphisms predispose to NSAID-induced PUD.

A recent preliminary study by Mallah et al. identified polymorphisms in platelet activity, angiogenesis and inflammatory response genes were associated with aspirin-related UGIB (Mallah and et al., 2020), building on the findings of previous investigations (Shiotani et al., 2010; Shiotani and et al., 2013; Shiotani et al., 2014; Wu et al., 2016; Cho and et al., 2016; Milanowski et al., 2017). The group identified “positive markers,” that indicated an increased risk of aspirin related UGIB and protective “negative markers” which decreased the risk (Mallah and et al., 2020). Of particular interest, rs689466 T > C, a SNP in the *COX-2* gene was associated with an increased risk of UGIB (Mallah and et al., 2020). However, these data are preliminary and need to be replicated in other cohorts.

There is significant literature describing associations between NSAID-induced immune-mediated ADRs and HLA alleles. These associations are with type I (immediate) urticarial and anaphylactic reactions (*HLA-DR11* and aspirin (Quiralte et al., 1999) and also type IV (delayed) reactions such as Stevens-Johnson syndrome/toxic epidermal necrolysis (HLA-B*73:01 and oxicam (Lonjou et al., 2008)). To the best of our knowledge, there are no reports of HLA associations with NSAID-induced GI toxicity which is reflective of the non-immune mediated nature of the adverse event.

Based on the pharmacogenomic data presented herein, it is reasonable to suggest that evidence for genetic predisposition to NSAID-induced upper GI toxicity is somewhat contradictory. This reflects the complex nature of the etiology of this ADR but also the significant inter-individual variability in non-genetic risk factors. There are a number of factors which may influence inter-individual pre-disposition to NSAID GI and need to be integrated with pharmacogenomics to further our understanding of individual risk. These include, but not limited to: 1) the impact of altered GI physiology and subsequent drug disposition in special groups and the impact of disease physiology (Stillhart et al., 2020), 2) variability in physiological regulation via gastric enteroendocrine cells (Mace et al., 2015) and 3) *Helicobacter pylori* infection which is a significant risk factor for peptic ulceration independent of NSAID use (Huang et al., 2002).

## Beyond the Human Genome

Spurred on by the completion of the Human Microbiome Project (2012), growing evidence supports the role of the microbiome in health and disease. Detrimental changes in gut microbiota composition (intestinal microbiota dysbiosis) are suggested as markers of GI pathogenesis in Crohn’s disease (Gevers et al., 2014), UC (Shen et al., 2018), NSAID-induced enteropathy (Montalto et al., 2013; Syer and Wallace, 2014; Rekatsina et al., 2020) and ulcer healing (Blackler et al., 2015). Microbiome heterogeneity may also influence drug response (Structure, function and diversity of the healthy human microbiome, 2012; Forslund et al., 2015; Wu et al., 2017; Ma et al., 2019; Sharma et al., 2019; Doestzada et al., 2018; Zimmermann et al., 2019; Yip and Chan, 2015; Kashyap et al., 2017).

NSAID disposition, therapeutic efficacy and toxicity are influenced by dynamic and complex host-gut microbiota interactions. Gut microbiota may directly modify NSAID chemistry or manipulate host metabolic processes affecting drug pharmacokinetics and pharmacodynamics. NSAIDs may modify gut microbiota composition resulting in dysbiosis (Maseda and Ricciotti, 2020). Rogers and Aronoff (2016) reported differences in gut microbiota profiles between non-users and NSAID-users. Gut microbiome composition also varied with the type of NSAID ingested (Rogers and Aronoff, 2016).

The capacity to intentionally manipulate the microbiota through diet (Dzutsev et al., 2017; Thiele et al., 2017; Ma et al., 2019), antibiotics/antimicrobials (Lanas and Scarpignato, 2006), fecal microbiota transplant (FMT) (Garber, 2015) or administration of selected probiotic strains (Gionchetti et al., 2000; Ulisse et al., 2001; Gionchetti et al., 2003; Montalto et al., 2010; Montalto et al., 2013; Suzuki et al., 2017; Mortensen et al., 2019; Rekatsina et al., 2020) to enhance efficacy and preserve functional mucosal integrity provides potential therapeutic value for various GI diseases. Recent developments in metaproteomics-based assays offer novel insights into microbiome absolute abundance and functional responses to drugs (Li and et al., 2020). Deployment of next generation sequencing technologies to identify predictive, diagnostic, and prognostic biomarkers need to be further investigated to determine their role in NSAID-induced GI pathology.

## Future Opportunities and Clinical Implementation

Further research into genetic risk factors predisposing individuals to NSAID-induced upper GI toxicity is justified. Knowledge gaps remain. Currently, there is a shortfall of genome-wide studies, with candidate gene approaches dominating. Lack of consensus and scarcity of independent replication to validate findings has been problematic in published studies. Given the burden and prevalence of NSAID-induced GI ADRs, this is perplexing. A genome-wide approach would allow for unbiased identification of common and rare variants in both PK and PD related genes but would require a large sample size with detailed phenotyping of cases and controls. Indeed, a variability in phenotype definitions is a further challenge in identifying tractable genetic associations. Thus, focused collaborative efforts to standardize phenotype definitions, as shown with other ADRs (Carr et al., 2017; Pirmohamed et al., 2011; Alfirevic et al., 2014; Nicoletti and et al., 2020; Aithal et al., 2011; Behr et al., 2012; Pirmohamed et al., 2011) should be encouraged, drawing inspiration from an adapted ‘consensus approach’ (Pirmohamed et al., 2011).

## Conclusion

The pathogenesis of NSAID-induced GI toxicity is complex. PGx is a useful tool to improve pharmacotherapy, and aims to shift the paradigm of dosing regimens being extrapolated to entire populations (Jaccard et al., 2020). Interrogation of genetic factors which predispose individuals to NSAID-induced upper GI toxicity offers unquestionable benefit: pre-emptive testing minimizes the risks of ADRs, informing clinical practice guidelines and therapeutic recommendations to enhance pharmacotherapy (Macías et al., 2020; Theken et al., 2020). To date, the *CYP2C* locus has been shown to be important in some but not all studies. Taken together with the fact that some NSAIDs are only partially metabolized by CYP2C9, but still cause PUD, it can be concluded that *CYP2C* genetic variants are neither necessary nor sufficient to predispose to NSAID-induced PUD, but the presence of low activity *CYP2C9* alleles in a patient given a CYP2C9-metabolised NSAID may increase the risk of PUD. Further work however is also required to identify novel genetic predisposing factors using genome-wide approaches but this will require larger numbers of well phenotyped patients.

Another factor to consider is that as our population continues to age, the prevalence of multimorbidity will increase (Uijen and van de Lisdonk, 2008), which will be accompanied by polypharmacy. Multimorbidity and polypharmacy increase the likelihood of drug- and gene-based interactions (Turner and et al., 2020). The elderly are liable to be prescribed NSAIDs, including LDA, because of the high prevalence of cardiovascular disease and arthritis. The risk of drug-drug interactions with concomitantly administered drugs is likely to be increased in the elderly: these may occur at both pharmacokinetic, (e.g. inhibition of CYP2C9 metabolism) and pharmacodynamic, (e.g. use of NSAIDs and corticosteroids) levels, and may thus contribute to the increased risk of upper GI toxicity. Thus with prolific NSAID use in the over 65 year olds, combinations of risk factors will be common and cumulative, increasing the risks of NSAID-induced ADRs (Laine et al., 2002). It is likely that these ADRs will increase in prevalence despite the use of gastroprotective therapies such as proton pump inhibitors.

In summary therefore, there is a continuing need to define genetic predisposing factors for NSAID-induced PUD because NSAIDs are very widely used, the occurrence of PUD is associated with a high degree of morbidity and mortality, and it is likely with the change in our age demographics, the problems of NSAID-induced PUD are likely to increase rather than decrease.

## Abbreviations

AA, Arachidonic acid; ADR, Adverse drug reaction; ASA, (Acetylsalicylic acid) Aspirin; COX, cyclo-oxygenase; Coxibs, COX-2 inhibitors; CPIC, Clinical Pharmacogenetics Implementation Consortium; CV, Cardiovascular; CYP, Cytochrome P450; FMT, Fecal microbiota transplant; GI, Gastrointestinal; GWAS, Genome wide association study; IBD, Inflammatory bowel disease; LD, Linkage disequilibrium; LDA, Low dose aspirin; NSAID, Non-steroidal anti-inflammatory drug; OTC, Over-the-counter; PG, Prostaglandin; PGx, Pharmacogenomics; PhV, Pharmacovigilance; PUD, Peptic ulcer disease; SNP, Single nucleotide polymorphism; TX, Thromboxane; tNSAID, Traditional NSAID; UC, Ulcerative colitis; UGIB, Upper gastrointestinal bleeding; UGT, Uridine 5′-diphosphate glucuronosyltransferases (UDP-glucuronosyltransferase).
